# A Clinical Audit on the Indications for Intervention With Transcatheter Aortic Valve Implantation Over Surgical Aortic Valve Replacement in Aortic Stenosis Patients in Mohammed Bin Khalifa Bin Salman Al Khalifa Specialist Cardiac Centre

**DOI:** 10.7759/cureus.39249

**Published:** 2023-05-19

**Authors:** Marya Al-Hammadi, Latifa Fakhroo, Nazar Bukamal

**Affiliations:** 1 Anaesthesia, King Hamad University Hospital, Muharraq, BHR; 2 General Practice, Royal College of Surgeons in Ireland, Busaiteen, BHR; 3 Consultant Cardiothoracic Anaesthesia and Critical Care, Mohammed Bin Khalifa Bin Salman Al Khalifa Specialist Cardiac Centre, Awali, BHR

**Keywords:** quality improvement research, esc/eacts guidelines, clinical audit, aortic stenosis (as), surgical aortic valve replacement (savr), transcatheter aortic valve implantation (tavi)

## Abstract

Introduction

Transcatheter aortic valve implantation (TAVI) is a novel treatment strategy used to treat patients with symptomatic aortic stenosis. It utilizes a percutaneous approach and is preferred over surgical aortic valve replacement (SAVR) in patients at high surgical risk. The aim of this study was to audit the indications of the intervention with TAVI over SAVR in Bahrain Defence Force Hospital, Mohammed Bin Khalifa Bin Sulman AlKhalifa Cardiac Centre (BDF-MKCC), as well as note the outcomes of patients who underwent TAVI.

Methods

The indications for allocating aortic stenosis patients to TAVI over SAVR in BDF-MKCC were studied with regard to the European Society of Cardiology and the European Association for Cardio-Thoracic Surgery (ESC/EACTS) guidelines published in 2017. Data from 82 patients, which accounts for all patients who underwent TAVI, were collected retrospectively from electronic medical records and the percentage of compliance was calculated and analyzed.

Results

The compliance percentages of the 23 parameters for the intervention with TAVI that have been set by the ESC/EACTS are calculated, where BDF-MKCC were fully adherent to 12 out of the 23 standards. Moreover, the total number of patients that are compliant with all standards is 13 out of 82 (15.85%) compliant patients.

Conclusion

The centre showed non-compliance to many of the published standards. Hence, we created a checklist to ensure that the international guidelines are followed. We are looking forward to re-audit this aspect in the near future, to make certain that changes were done. We would also like to do a comparative study to compare the patients’ outcomes before and after implementing the 2017 ESC/EACTS guidelines. Furthermore, we call for further studies to be conducted in this field and that is to evaluate the standards themselves as well as the safety of TAVI in those who are not eligible for it according to the ESC/EACTS.

## Introduction

Aortic stenosis (AS) is defined as constriction of the aortic valve orifice, most commonly occurring due to degeneration and subsequent fibrosis and calcification of the valve's leaflets [1]. AS has a significant effect on the morbidity and mortality of patients and that is through increasing the pressure afterload on the left ventricle and the resulting succeeding cascade of events such as ventricular hypertrophy [1]. A population-based study done on 2000 US adult patients suggests an incidence of 0.4% of AS in the general population, most of which were adults 75 years of age and older [2]. Moreover, a prospective survey of patients with valvular heart disease in Europe also revealed that the most common native valve disease is AS, which particularly affected the elderly with comorbidities [2-4].

There are various ways to manage AS; including surgical intervention and percutaneous approaches [1,5]. Surgical aortic valve replacement (SAVR) remains the gold standard therapy for patients with AS [1,5]. Furthermore, examples of percutaneous therapies include balloon valvuloplasty which involves the inflation of a balloon to dilate the aortic valve, percutaneous valve implantation, also termed transcatheter aortic valve implantation (TAVI), which is a novel treatment strategy that emerged in the past few decades and is now being used increasingly, as it acts as a less invasive alternative treatment especially benefiting those at high surgical risks, as well as other percutaneous therapies [1,5-7].

The first prosthetic valve implantation in humans was performed by Pr. Alain Cribier, in 2002, on a 57-year-old male with AS [8]. According to National Institute for Health and Care Excellence (NICE) guidelines, TAVI can be simply described as orthotopic implantation of a valve which can be done through different access routes: a transluminal route, which utilizes large arteries usually the femoral or subclavian artery, or the surgical approach, much less commonly used, which involves a mini-thoracotomy with a transapical approach, the decision of which is made depending on various factors and preferences [9]. The transfemoral approach is the most frequently used route [6].

In 2017, the European Society of Cardiology and the European Association for Cardio-Thoracic Surgery (ESC/EACTS) published international guidelines to aid the decision-making of cardiologists when it comes to AS management [10]. These guidelines came into force following considerable studies that reviewed TAVI patients [10].

Like all therapeutic procedures, and despite its success as an alternative treatment strategy, TAVI is associated with post-procedural complications, such as [11,12], procedure failure for instance valve embolization, stroke, aortic regurgitation, coronary ostia obstruction, bleeding, arrhythmias that may require pacemakers and death. Nevertheless, outcomes remain favourable in comparison with high-surgical-risk patients who underwent SAVR [13].

Bahrain Defence Force Hospital, Mohammed Bin Khalifa Bin Sulman AlKhalifa Cardiac Centre (BDF-MKCC) implemented TAVI in February 2014, and being the only cardiac centre in the Kingdom of Bahrain makes them the first in the country to practice this technique. In this paper, we will be auditing the indications that have led to the use of TAVI over SAVR in BDF-MKCC and compare them to the ESC/EACTS guidelines, as well as noting the outcomes of patients who underwent TAVI.

## Materials and methods

In this retrospective clinical audit, we studied the indications for the intervention of 82 patients diagnosed with severe AS who have undergone TAVI, authorized by the multidisciplinary team at MKCC.

All patients who underwent TAVI were assessed from the implementation of the practice in February 2014 until December 2018 (the commencement of this clinical audit). No inclusion or exclusion criteria were required, thus preventing selection bias. Ethical approval was sought from BDF ethical committee.

The patient’s medical records were collected from three databases used by BDF-MKCC; the electronic medical records (EMR); the Royal Medical Services (RMS) applications, the radiology software JIVEX and the Apollo software from which the echocardiography reports were reviewed. All patients’ data and statistical analysis will be explored in an Excel software spreadsheet.

The Online Society of Thoracic Surgery (STS) Adult Cardiac Surgery risk calculator [14] was used to calculate the percentage risk of mortality, the Charlson Comorbidity Index [15] to calculate the percentage estimated 10 years of survival, and the Clinical Frailty Scale [16] to assess the overall fitness and frailty of the patients based on clinicians' evaluation.

Moreover, the 30-day and one-year post-TAVI intervention outcomes were evaluated.

Our objective in this retrospective clinical audit study is to appraise the BDF-MKCC's decision-making in intervening with TAVI according to the ESC/EACTS international guidelines [10] since this procedure is novel and has recently been introduced to the centre. Table 1 summarizes the indications to choose TAVI over SAVR [10].

**Table 1:** The ESC/EACTS guidelines ESC/EACTS: European Society of Cardiology and the European Association for Cardio-Thoracic Surgery, SAVR: surgical aortic valve replacement, TAVI: transcatheter aortic valve implantation, LV: left ventricle, CAD: coronary artery disease, CABG: coronary artery bypass grafting

ESC/EACTS guidelines	
1	STS/EuroSCORE II ≥4%
2	Presence of severe comorbidity
3	Age ≥ 75 years
4	Previous cardiac surgery
5	Frailty
6	Restricted mobility and conditions that may affect the rehabilitation process after the procedure
7	Suspicion of endocarditis (favours SAVR)
8	Favourable access for transfemoral TAVI
9	Sequelae of chest radiation
10	Porcelain aorta
11	Presence of intact coronary bypass grafts at risk when sternotomy is performed
12	Expected patient-prosthesis mismatch
13	Severe chest deformation or scoliosis
14	Short distance between coronary ostia and aortic valve annulus out of range for TAVI (favours SAVR)
15	Size of aortic valve annulus out of range for TAVI (favours SAVR)
16	Aortic root morphology unfavourable for TAVI (favours SAVR)
17	Valve morphology (bicuspid, degree of calcification, calcification pattern unfavourable for TAVI
18	Presence of thrombi in aorta or LV (favours SAVR)
19	Severe CAD requiring revascularization by CABG (favours SAVR)
20	Severe primary mitral valve disease, which could be treated surgically (favours SAVR)
21	Severe tricuspid valve disease (favours SAVR)
22	Aneurysm of the ascending aorta (favours SAVR)
23	Septal hypertrophy requiring myectomy (favours SAVR)

## Results

The data of all 82 TAVI patients were collected and analysed with regard to the ESC/EACTS guidelines. The outcomes were also noted. The mean age of this population is 76, ranging from 56 to 98 years of age. Additionally, the male and female distribution is 38 (46.3%) and 44 (53.7%) respectively.

Table 2 demonstrates the adherence rate of BDF-MKCC to the ESC/EACTS standards that allocate patients to TAVI over SAVR.

**Table 2:** Compliance with the indication standards for intervention with TAVI in patients with AS TAVI: transcatheter aortic valve implantation, STS: STS score, LV: left ventricle, CAD: coronary artery disease, CABG: coronary artery bypass grafting; AS: aortic stenosis

	Standards	No. of patients (n=82)	Compliance with TAVI (%)
1	STS ≥4%	26	31.70%
2	Presence of severe comorbidity	68	82.93%
3	Age ≥ 75 years	49	59.80%
4	Frailty	65	79.30%
5	Restricted mobility and conditions that may affect the rehabilitation process after the procedure	65	79.30%
6	No suspicion of endocarditis	82	100%
7	Favourable access for transfemoral TAVI	80	97.56%
8	Distance between coronary ostia and aortic valve annulus within the range for TAVI	82	100%
9	Size of aortic valve annulus within the range for TAVI	82	100%
10	Aortic root morphology favourable for TAVI	82	100%
11	Valve morphology favourable for TAVI	81	98.78%
12	Absence of thrombi in aorta or LV	82	100%
13	No severe CAD requiring revascularization by CABG	78	95.10%
14	Absence of severe primary mitral valve disease, which could be treated surgically	82	100%
15	Absence of severe tricuspid valve disease	77	93.90%
16	Absence aneurysm of the ascending aorta	82	100%
17	Absence of septal hypertrophy requiring myectomy	82	100%

On the other hand, Table 3 shows the adherence rate in special cases. According to the ESC/EACTS, having any of these cases should favour TAVI over SAVR. All patients presenting with these cases were assessed and the decision of therapy was noted and analysed.

**Table 3:** Compliance with TAVI standards in special cases TAVI: transcatheter aortic valve implantation, SAVR: surgical aortic valve replacement, AS: aortic stenosis

	Special cases	TAVI (n=82) SAVR (n=230)		Patients with AS (n=312)	Compliance with TAVI (%)
1	Previous cardiac surgery	TAVI	16	25	64%
		SAVR	9		
2	Sequelae of chest radiation	TAVI	3	3	100%
		SAVR	0		
3	Porcelain aorta	TAVI	2	2	100%
		SAVR	0		
4	Presence of intact coronary bypass grafts at risk when sternotomy is performed	TAVI	14	23	60.87%
		SAVR	9		
5	Expected patient-prosthesis mismatch	TAVI	0	0	-
		SAVR	0		
6	Severe chest deformation or scoliosis	TAVI	2	2	100%
		SAVR	0		

Furthermore, Table 4 lists the outcomes of patients who underwent TAVI.

**Table 4:** Outcomes of patients who underwent TAVI TAVI: transcatheter aortic valve implantation

Outcomes	No. of patients (n=82)		
	30-day		1-year
Stroke	1 (1.22%)		0 (0%)
Femoral artery injury required surgical repair	2 (2.43%)		N/A
Femoral artery dissection or stenosis required stent graft	2 (2.43%)		N/A
Femoral artery dissection or stenosis not requiring treatment	1 (1.22%)		N/A
Aortic insufficiency (vale-in-valve)	4 (4.89%)		0 (0%)
Mortality	0 (0%)		6 (7.32%)
Chronic heart block required permanent pacemaker	-	11 (13.41%)	

## Discussion

Looking at each standard, BDF-MKCC were adherent to some standards but showed non-compliance to others. Out of the 82 patients, compliance with international standards was seen in 13 cases only. Therefore, BDF-MKCC should adopt the newest version of ESC/EACTS guidelines, published in 2017 [10].

Each standard cannot independently stratify patients into the high-surgical-risk category, the ESC reports that they should be appraised together and in conjunction with other characteristics such as clinical judgment and presence of contraindications to TAVI to ensure optimal decision-making [10,17]. Therefore, the guidelines put together all the essential standards requiring consideration when selecting patients for TAVI [10,17]. The standards to which BDF-MKCC were not compliant, along with their significance and justification, will be discussed in this section.

Firstly, the STS risk score is a tool used to estimate the mortality-risk, and other endpoints, following valve surgery, however, lately, it is being adequately utilized for transcatheter procedures [18]. Assessing the STS score can, independently, guide therapeutic decision-making and predict the prognosis of the intervention [19]. SAVR remains the gold standard therapy for patients with AS [1,20]. Nonetheless, TAVI, being a less invasive procedure, is prioritized for patients at high surgical risk [7]. Therefore, ESC/EACTS chose to allocate patients with low and high surgical risk, to SAVR and TAVI respectively. BDF-MKCC were non-compliant with this standard due to the following reasons; one patient had sickle cell disease and blood crossmatch was difficult, six patients were private patients that requested TAVI, 10 patients refused SAVR, 18 cases were requested by a Saudi Arabian board, leaving 20 patients that were allocated to TAVI based on clinical judgement. Barbash et al. proved that choosing TAVI for low- and intermediate-risk patients is safe, moreover, the outcomes were improved compared to patients at high risk [21]. Meanwhile, O’Brien et al. reported that STS scores were higher in death cases of TAVI [22]. Consequently, Voigtländer et al. suggest lowering the STS score threshold, such that patients with a score ≤4% are also assigned to TAVI, though, it was stated that some areas are yet to be studied to ensure the accuracy of such decision [23].

Moving on to age, many TAVI patients in BDF-MKCC were <75 years of age as opposed to the ESC/EACTS recommendations. After reviewing the literature, studies proved that age does not influence the outcomes of TAVI significantly, however, the aim of these studies was to ensure the safety of TAVI in older patients rather than its effectiveness in younger, low-risk patients [24,25]. Eggebrecht et al. [26] decided to focus on younger patients instead, and he reported that looking at the outcomes, TAVI was not inferior to SAVR when it comes to complications such as post-operative delirium or needs for dialysis, although patients undergoing TAVI were more likely to require new pacemakers post-operatively and proposed the possibility of them experiencing technical issues in the long run. Therefore, he calls for trials to study the efficiency of TAVI in terms of long-term outcomes [26].

Frailty can be used to predict the prognosis and mortality post-TAVI [27]. On that account, TAVI is prioritized for frail patients as this factor is a contributor to stratifying patients into a high-surgical-risk category [17]. In this study, an assessment of patients' frailty by done using the Rockwood clinical frailty scale, which takes into account the mobility, function and cognition of the patient, thus all frail patients are considered to have a degree of restricted mobility [28,16]. A meta-analysis study exploring the relationship between preoperative frailty and outcomes reported an increase in mortality in frail individuals (34 deaths per year) compared to non-frail individuals (19 death per year) [29]. Likewise, the presence of severe co-morbidities is proven to prognosticate patients undergoing surgical interventions [30]. BDF-MKCC did not introduce the frailty or comorbidity assessment in their patient selection criteria, hence, suboptimal adherence to these standards was reflected. Similar to expanding the STS range, there are challenges that face in making TAVI the first-line therapy for AS, and these include long-term valve durability, the need for predictable results such as rate of vascular complications, paravalvular leakage and a new need for permanent pacemakers, all of which require further studying and evidence, the NOTION-2 trial is a randomized controlled trial being conducted to study the areas discussed, however to date, no evidence is available [31].

BDF-MKCC was not compliant with the transfemoral approach standard in two cases, where the subclavian route was sought. The transfemoral route remains the first-line approach for TAVI, especially since it entails the least invasive techniques [32]. Nevertheless, in some cases, the transfemoral route is rendered unsuitable, like a tortuous or sclerotic femoral artery. For instance, in our study, both patients had small, tortuous femoral arteries, therefore, the subclavian route was approached. The subclavian route provides a decent alternative to the conventional route, and outcomes revealed it to be a safe approach, yet, due to the longer procedure duration and absence of advantages over the transfemoral approach, it is only used for selected patients [33].

With early-generation devices, outcomes of TAVI in patients with bicuspid aortic valve (BAV) are suboptimal compared to tricuspid aortic valves (TAV), however, with the emergence of new-generation devices, outcomes are improving [34]. The ESC guidelines, therefore, suggest that only those with TAV morphology are fit for TAVI. In BDF-MKCC, one patient had a BAV, hence proving non-adherence to this standard as the patient was referred by a Saudi committee (ARAMCO). The significance of adhering to this standard can be rationalized through studies reporting the consequences of TAVI in patients with BAV. William et al. reported that the left ventricular outflow tract in patients with BAV is significantly larger than those with TAV [35], which explains why these patients are prone to paravalvular leaks, other outcomes associated with BAV include stroke and the need for new pacemaker [34]. The patient in our study developed severe aortic insufficiency postoperatively.

Commonly, CAD and severe AS coexist, moreover, studies reported that the presence of CAD in patients undergoing TAVI increases the risk of mortality, stroke and myocardial infarction [36,37]. BDF-MKCC did TAVI on four patients with severe CAD, hence showing non-compliance to this standard. All four refused surgery, so TAVI was performed according to the patient’s choice and following TAVI, percutaneous coronary intervention (PCI) was done to reduce the risk of complications.

According to Baumgartner, the presence of severe tricuspid valve disease is associated with the worst clinical outcomes in patients undergoing TAVI [38]. However, as stated by the ESC these patients should be treated surgically, where studies reported a 50% chance of improvement of tricuspid regurgitation (TR) after SAVR, compared to 15-50% of TAVI [39,40]. Despite the limitations, the BDF-MKCC committee took the risk and enrolled five patients with severe TR for TAVI and decided to monitor TR post-TAVI.

When assessing all patients with previous cardiac surgery, including CABG, some cases in this centre were assigned for SAVR rather than TAVI which demonstrates a lack of adherence to these ESC standards. Although a meta-analysis reported that post-operative outcomes like stroke and bleeding are higher in SAVR patients, it also reports that STS score has to be accounted for, where patients with low STS score are safe to undergo SAVR [41]. In our case, all nine candidates had low STS scores and thus were allocated to SAVR by the centre.

After noting the outcomes of TAVI patients in BDF-MKCC, none expired within 30 days, however, six cases expired within one year post-TAVI due to non-cardiac aetiologies; one patient died of cancer, one patient died of sepsis, renal failure and arrest, another patient expired due to intracranial haemorrhage, one patient died of complicated chest infection, and lastly two patients died of sepsis secondary to an abdominal aetiology. All remaining outcomes, listed in Table 4, developed within 30 days post-TAVI.

There are a few limitations to our study design. Firstly, being a retrospective study, some data were missing due to inadequate documentation and therefore some information was acquired from other parties such as the treating cardiologist. Furthermore, on inquiry, we ensured that the centre calculated the STS scores for all patients but the scores were seldom documented. Moreover, on conducting a literature review, we conclude that there are multiple definitions for frailty and no established guidelines for frailty scoring. In this study, the Rockwood clinical frailty scale was chosen as suggested to be adequate by Juma et al. [42].

**Figure 1:**
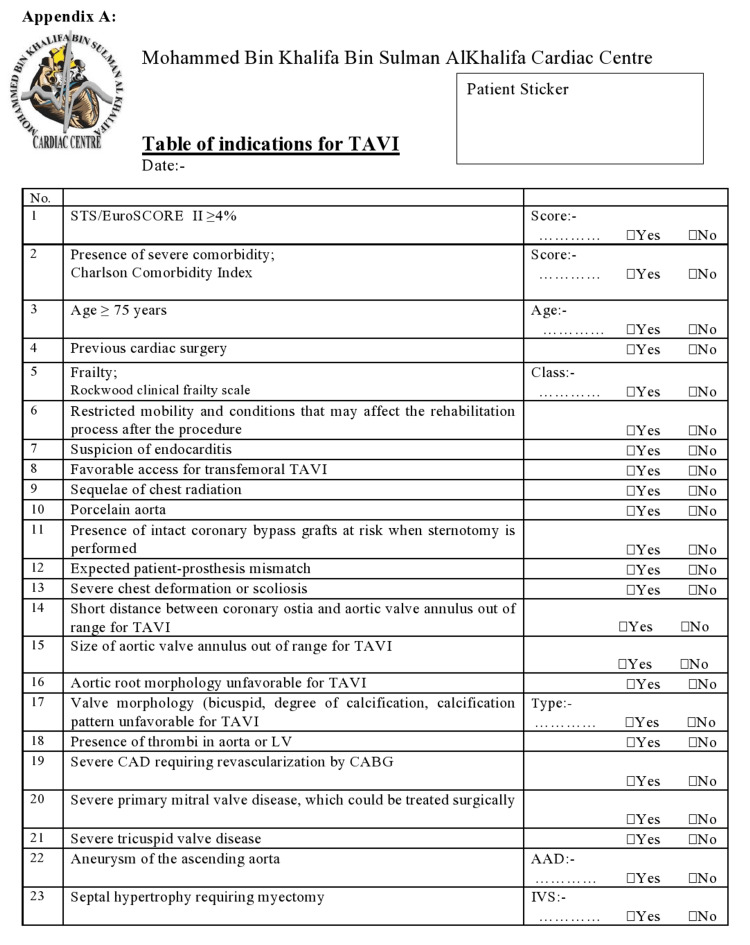
Checklist of indications of transcatheter aortic valve implantation

**Figure 2:**
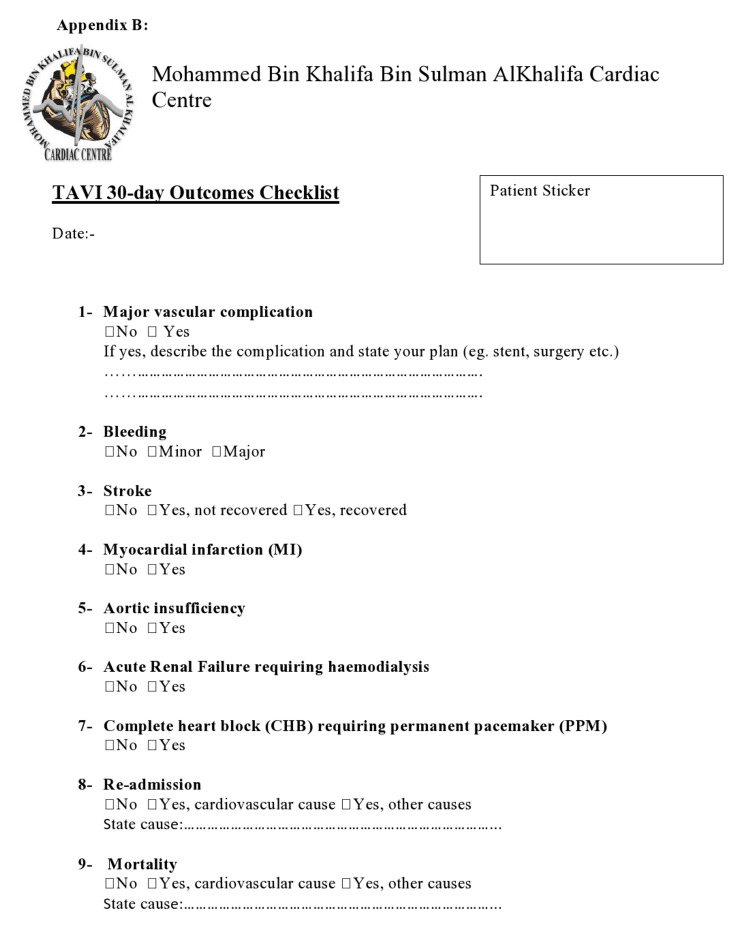
Transcatheter aortic valve implantation 30-day outcomes checklist

**Figure 3:**
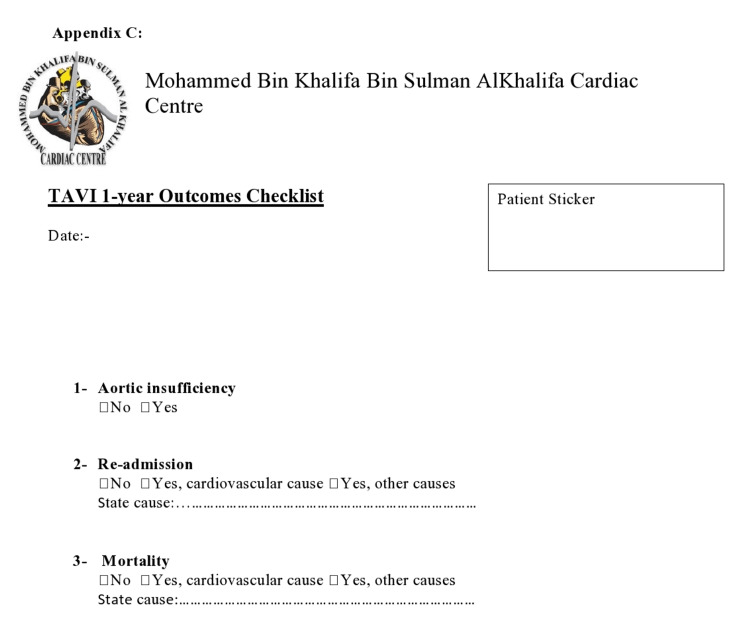
Transcatheter aortic valve implantation one-year outcomes checklist

Hence, to increase the efficiency of BDF-MKCC when it comes to allocating patients to TAVI, three forms, designed to be filled electronically and be part of patients’ EMR, were created. The first of these forms, the most important, is a checklist listing all 23 standards suggested by the ESC/EACTS to ensure proper decision-making (Appendices, Figure 1). Moreover, two checklists listing possible outcomes 30 days and one-year post-TAVI development were created (Appendices, Figure 2 and Figure 3).

## Conclusions

In conclusion, this clinical audit assessed the indications for intervention with TAVI over SAVR on patients with severe AS, using the ESC/EACTS guidelines as the international standard for intervention. The guidelines constitute 23 standards and BDF-MKCC was 100% compliant with 12 standards only. Moreover, out of the 82 patients, BDF-MKCC were compliant with international standards in 13 cases only. Thus, we created a checklist to ensure that the centre follows international guidelines. We are looking forward to re-audit this area in the near future, to make certain that changes were done. We would also like to do a comparative study to compare the patients' outcomes before and after implementing the 2017 ESC/EACTS guidelines. Furthermore, we call for further studies to be conducted in this field to evaluate the standards themselves as well as the safety of TAVI in those who are not eligible for it according to the ESC/EACTS.
